# Prediction of conformational B-cell epitopes from 3D structures by random forests with a distance-based feature

**DOI:** 10.1186/1471-2105-12-341

**Published:** 2011-08-17

**Authors:** Wen Zhang, Yi Xiong, Meng Zhao, Hua Zou, Xinghuo Ye, Juan Liu

**Affiliations:** 1 School of Computer, Wuhan University, Wuhan 430072, China

## Abstract

**Background:**

Antigen-antibody interactions are key events in immune system, which provide important clues to the immune processes and responses. In Antigen-antibody interactions, the specific sites on the antigens that are directly bound by the B-cell produced antibodies are well known as B-cell epitopes. The identification of epitopes is a hot topic in bioinformatics because of their potential use in the epitope-based drug design. Although most B-cell epitopes are discontinuous (or conformational), insufficient effort has been put into the conformational epitope prediction, and the performance of existing methods is far from satisfaction.

**Results:**

In order to develop the high-accuracy model, we focus on some possible aspects concerning the prediction performance, including the impact of interior residues, different contributions of adjacent residues, and the imbalanced data which contain much more non-epitope residues than epitope residues. In order to address above issues, we take following strategies. Firstly, a concept of 'thick surface patch' instead of 'surface patch' is introduced to describe the local spatial context of each surface residue, which considers the impact of interior residue. The comparison between the thick surface patch and the surface patch shows that interior residues contribute to the recognition of epitopes. Secondly, statistical significance of the distance distribution difference between non-epitope patches and epitope patches is observed, thus an adjacent residue distance feature is presented, which reflects the unequal contributions of adjacent residues to the location of binding sites. Thirdly, a bootstrapping and voting procedure is adopted to deal with the imbalanced dataset. Based on the above ideas, we propose a new method to identify the B-cell conformational epitopes from 3D structures by combining conventional features and the proposed feature, and the random forest (RF) algorithm is used as the classification engine. The experiments show that our method can predict conformational B-cell epitopes with high accuracy. Evaluated by leave-one-out cross validation (LOOCV), our method achieves the mean AUC value of 0.633 for the benchmark bound dataset, and the mean AUC value of 0.654 for the benchmark unbound dataset. When compared with the state-of-the-art prediction models in the independent test, our method demonstrates comparable or better performance.

**Conclusions:**

Our method is demonstrated to be effective for the prediction of conformational epitopes. Based on the study, we develop a tool to predict the conformational epitopes from 3D structures, available at http://code.google.com/p/my-project-bpredictor/downloads/list.

## Background

Within an immune system, antigen-antibody (Ag-Ab) interaction plays a critical role in the immune processes and responses, and the sites on antigens that are recognized and bound by B cell-produced antibodies are well known as B-cell epitopes [1]. B-cell epitopes can be used to synthesize peptides that elicit the immune response with specific cross-reacting antibodies [2,3]. For this reason, the identification of B-cell epitopes becomes a critical component of epitope-based vaccine design. B-cell epitopes can be categorized into two types: linear (continuous) epitopes and conformational (discontinuous) epitopes. Linear epitopes comprise residues that are continuous in the sequence, while conformational epitopes consist of residues that are distantly separated in the sequence but have spatial proximity. The wet experiment for the epitope identification is time-consuming, labor-intensive, and expensive. With increasing availability of experimentally derived epitopes, it becomes possible to develop computational methods for epitope prediction [4], which are faster and more economical.

In the past, researchers had been focusing on the prediction of linear epitopes. The classic way of predicting linear B-cell epitopes is based on amino acid propensities [5-10]. These commonly used propensities are hydrophilicity scale, flexibility scale, surface accessibility scale, exposed residue scale, beta-turn scale, antigenicity scale, polarity scale and so on. However, these methods are proved to be marginally better than random models [11]. Subsequently, various machine learning methods were introduced into B-cell epitope prediction, such as HMM [12], decision tree [13], nearest-neighbor method [13], ANN [14] and SVM [15-17]. The machine learning-based models can well describe the nonlinear relationship between propensities and the location of linear epitopes, and thus lead to the improved performance. However, these linear epitope prediction methods cannot be used to predict conformational epitopes, which take majority of the epitopes.

A limited number of methods have been proposed for the conformational epitope prediction. Unlike the linear epitopes that are usually determined by the linear peptide segments, the conformational epitopes are mostly influenced by spatial adjacent regions. The locations of epitopes are often considered to be correlated with some physicochemical and structural features of spatial adjacent regions. CEP used the 'solvent accessibility' of amino acids to identify epitopes [18]. DiscoTope combined the surface accessibility, spatial information and amino acid statistics information to distinguish epitopes from non-epitope regions [19]. PEPITO was proposed by combining amino-acid properties and half sphere exposure values at multiple distances [20]. ElliPro is a web-tool that is based on Thornton's method and a residue clustering algorithm [21]. In SEPPA [22], two concepts 'unit patch of residue triangle' and 'clustering coefficient' were introduced to describe the local spatial context and spatial compactness. Moreover, some protein-protein docking methods such as PatchDock [23] and ClusPro [24] can be used for the epitope prediction as well.

Recently, the spatial context of an antigen residue is usually described by a concept of 'surface patch', which consists of some spatially nearest surface residues and the considered residue itself (named 'central residue' of the patch). The patches can be classified into two types, the non-epitope patch and the epitope patch, according to the states of the central residues (non-epitope or epitope). Thus, the epitope prediction can be formulated as a binary classification problem (or regression in some methods). By using the surface patch, some machine learning methods have been applied to the conformational epitope prediction. EPITOPIA used several structural and physicochemical features to represent the surface patch [25,26], and adopted the naive Bayes classifier to make predictions. EPCES [27] introduced a consensus scoring method based on different structural and physicochemical terms. By using similar features, EPSVR [28] adopted the SVM regression to make predictions. EPMeta [28] is a meta model that ensembles the results from several existing prediction servers. Liu used the logistic regression to predict the conformational epitopes based on the structural information [29]. In addition to the structure-based methods, a sequence-based method is recently proposed to predict the conformational epitopes [30].

Although several methods were proposed for conformational epitope prediction, the reported performance is far from satisfaction. There are some possible points concerning the epitope prediction performance: (1) for antigens, there are much more non-epitope residues than epitope residues; (2) the spatial characteristics of the epitopes is usually described by the surface patch, which consists of adjacent surface residues, but interior residues are not included in the patch or evaluated; (3) the residues in a patch may make different contributions to the location of epitopes, and the different contributions should be quantitatively represented.

In order to design the optimal model, we take following strategies. Firstly, we propose a novel concept named 'thick surface patch' to describe the spatial characteristics of antigen residues, which include adjacent surface residues as well as interior residues. The study demonstrates the thick surface patch can yield better results than the surface patch, and indicates that adjacent interior residues indeed contribute to the recognition of conformational epitopes. Secondly, we observe the statistical significance of the distance distribution difference between epitope patches and non-epitope patches. Consequently, a distance-based adjacent residue distance feature (ARD) is proposed to differentiate the contributions of residues in a patch. Thirdly, a sophisticated bootstrapping and voting procedure is introduced to deal with the imbalanced dataset. Here, random forest [31] is used as the classification engine. Random forest algorithm has gained popularity in the bioinformatics community in recent years, successfully solving lots of similar problems, such as protein-protein binding site prediction and protein-DNA binding site prediction [32-36]. Based on above strategies, we develop a novel method for predicting B-cell conformational epitopes by using the random forest (RF) algorithm with the combination of the adjacent residue distance feature and several conventional features.

## Methods

### Datasets

Datasets used in the studies are relevant to their goals and scopes. Some conformational epitope prediction models are constructed on the bound structures, while others are built on the unbound structures. Therefore, we use both bound and unbound dataset to evaluate and compare models.

We use the dataset published by Rubinstein as the benchmark bound dataset [26]. The bound dataset consists of 66 non-redundant Ag-Ab structures, available at: http://epitopia.tau.ac.il/trainData/.

We use the Liang's dataset as the benchmark unbound dataset [28]. Liang's dataset is compiled as follows: (1) 22 antigen-antibody complexes and their unbound structures were sourced from protein docking Benchmark 2.0 [37]; 59 representative antigen-antibody complexes were provided by [38]; 17 antigen-antibody complex structures were collected from [27]; (2) these structures were merged, and the complexes without available unbound structure were removed. Finally, a total of 48 complexes and their unbound structures were retained as the benchmark unbound dataset, available at: http://sysbio.unl.edu/services/.

In addition, the independent test set compiled from entries of the Conformational Epitope Database (CED) [39] is used, which contains 19 antigen structures with annotated epitopes. This dataset is available at: http://sysbio.unl.edu/services/.

We compile a benchmark dataset of 83 antigen sequences from Rubinstein's structure dataset, available at http://code.google.com/p/my-project-bpredictor/downloads/list. Hence, we can fairly compare the sequence-based models with structure-based models.

### Epitope definition

There are several definitions ever used for the epitopes inferred from the X-ray structures of Ag-Ab complexes, such as the accessible surface area loss upon antibody binding or the distance between antigen residues and antibody residues. However, the study in [38] indicated that different epitope definitions are likely to give out similar results. Hence, we follow the commonly used distance-based definition. Specifically, an antigen residue separated from any antibody residue by a distance less than 4Å is defined as an epitope residue, and the distance between two residues is measured by the minimal Euclidean distance between the centers of any of their non-hydrogen atoms.

### Thick surface patch

A residue is defined as the surface residue, if its relative accessible surface area (RASA) calculated by DSSP program [40] is more than 5%. When using the surface patch to describe the spatial characteristics of antigen residues, the epitope residues and non-epitope residues are considered to be distinct with respect to their surface patches. We notice that the surface patch only include the surface residues, therefore this raises a question: are the adjacent interior residues unimportant or unnecessary for the representation of spatial context? Clearly, the interior residues cannot be epitope residues, but it does not mean that they cannot influence surface residues, and the interior residues may contribute to the formation of epitope sites. In order to address the issue, the impact of interior residues cannot be neglected and should be investigated. In this study, we propose a new concept 'thick surface patch'. Formally, the thick surface patch of a surface residue is defined as a set of *n *nearest adjacent residues, including interior neighbors as well as surface neighbors. For simplicity, the thick surface patch and the surface patch are generally named 'residue patch' in the following sections.

### Adjacent residue distance feature

The residue patch is critical for the conformational epitope prediction. However, contributions of residues in a patch may be distinct and depend on their distances to the central residue. Since existing methods usually used the patch of 20 residues, the analysis is implemented on the patch of this size.

**Figure 1:**
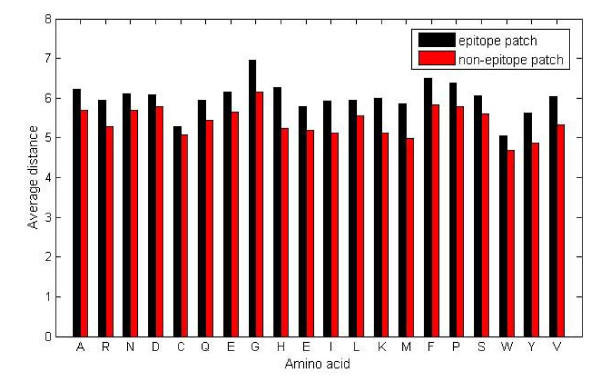
**Average distance (Å) between adjacent residues and the central residue in epitope patches versus non-epitope patches (for the bound dataset)**. X axis means the central amino acid type in the patch, and Y axis means average distance between adjacent residues and the central residue.

To test whether the distances between adjacent residues and the central residue have impact on the state of the central residue, we calculate the average distance between adjacent residues and the central residue, and the average distance is compared between epitope patches and non-epitope patches for each central residue type. The results reveal that non-epitope patches have significantly less average distance than epitope patches (P = 1.59 × 10^-10 ^by paired t-test for the bound dataset, see Figure 1).

**Figure 2:**
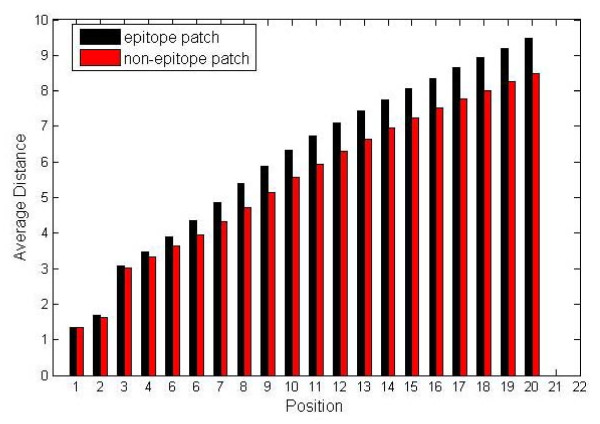
**Distance (Å) distribution in epitope patches versus non-epitope patches (for the bound dataset)**. X axis means *K*th nearest residues for a central residue (*k *= 1, 2, ..., 20). Y axis means average distance between *K*th nearest residues and the central residue.

For further test, we compare the distance between *k*th nearest adjacent residues (*k *= 1, 2... 20) and the central residue in epitope patches versus non-epitope patches. The results show that the distance distribution in epitope patches is significantly different from that of non-epitope patches (P = 1.20 × 10^-7 ^by paired t-test for the bound dataset, see Figure 2).

According to the statistical analysis on the bound dataset, it is observed that the average distance of the patch and the distance distribution of the patch may help to distinguish the epitope patches from non-epitope patches. The similar conclusion can be drawn for the unbound dataset (data not shown).

Based on the above study, we propose an adjacent residue distance feature based on the distance between the adjacent residue and the central residue, which is defined as follows:

$$S\left(x_{i}\right)=\frac{p_{i}}{\overset{n}{\underset{i=1}{\sum }}p_{i}}$$

Where *x_i _*represents an adjacent residue in a patch, *I *= 1, 2, ...,*n*. $p_{i}=\frac{1}{d_{i}}$, and *d_i _*is the Euclidean distance between *x_i _*and the central residue (based on the nearest non-hydrogen atoms). In the feature, the contributions of adjacent residues in a patch are quantitatively represented and depend on their relative distances to the central residue.

### Descriptors for residue patch

While constructing prediction models, each patch should be represented as a feature vector by using physicochemical and structural features. In addition to the adjacent residue distance feature, several popular physicochemical and structural features are used.

Relative accessible surface area: it is an important factor influencing the antigen-antibody binding, and the greater relative area of a surface residue means the greater probability of being an epitope residue. The relative accessible surface area of a residue is calculated by dividing its accessible surface area with the accessible surface area of fully exposed amino acid. The accessible surface areas of surface residues are calculated by using DSSP program [40], and the fully exposed amino acid area can be obtain from [41].

Evolutionary conservation: Generally speaking, functional regions on protein surfaces are usually more evolutionarily conserved than other regions, but the study on antigen crystal structures draws opposite conclusion. Statistical test reveals that evolutionary conservation can significantly distinguish epitopes from non-epitope region [42]. In order to calculate conservation scores, the primary sequence of the antigen chain we want to predict is aligned to the non-redundant protein database by using BLAST program (round of iteration is set to 3), and a position specific scoring matrix (PSSM) is returned. Then, the conservation score of the residue at the sequence position *i *is calculated by following function:

$$Score=\{\begin{array}{ccc} \left|M_{ir}-B_{rr}\right| & if & M_{ir}-B_{rr}<0\\ 0 & else & \end{array}$$

Here, *M_ir _*is the value of residue type *r *at the sequence position *i*, according to the PSSM, and *B_rr _*is the diagonal element of BLOSUM62 for residue type *r*. The same function is used in [28].

Secondary structure: secondary structures are proved as important factors for the Ag-Ab interaction, and epitopes are likely to have specific secondary structure elements versus non-epitope surfaces [42]. Here, we use DSSP to calculate the secondary structures of surface residues, and each secondary structure (helix, sheet or coil) is represented as a three-bit string, such as (1, 0, 0), (0, 1, 0) and (0, 0, 1), respectively.

Amino acid composition: amino acid composition is widely used in protein function analysis and classification. In the Ag-Ab interaction, some amino acid types are significantly overrepresented in epitopes, and others are underrepresented, thus the amino acid composition can be used to differentiate epitope patches from non-epitope patches [42]. For a patch, the percentage of each amino acid type is calculated as the amino acid composition.

With respect to these physicochemical and structural features, each residue in a residue patch can be represented as a feature vector of 7 dimensions (1 for relative accessible surface area, evolutionary conservation, the adjacent residue distance, and amino acid composition, respectively, 3 for secondary structure). As a result, a patch of *n *residues is represented by a 7 × *n *-dimensional feature vector.

### The strategy for the imbalanced dataset

In fact, a great number of real datasets are imbalanced, in which the instances from one class take majority of the data. The common machine learning methods cannot well handle the imbalanced dataset, and they are usually combined with some strategies to solve the problem. There are two common approaches to deal with the imbalanced datasets. One approach is assigning a high cost to the misclassification of minority class and redesigning the classifier by minimizing the error rate. The other is downsizing the majority class or upsizing the minority class.

An approach based on data bootstrapping and voting is used here to deal with the imbalanced data, summarized as follows,

1. Let *A *be the training set, *A*^- ^be the set of negative instances and *A*^+ ^be the set of positive instances, and there are much more negative instances than positive instances;

2. Random data sampling is implemented n times on the set *A*^- ^to obtain n data subset $A_{i}^{-}$ whose size is equal to the size of *A*^+^, *i *= 1, 2, ..., *n*;

3. Combined each $A_{i}^{-}$ and *A*^+ ^to generate *n *different training sets, *i *= 1, 2, ..., *n*, and a random forest model can be built on one training set. Totally, *n *models can be obtained;

4. Given a new instance, *n *random forest models (sub-classifier) will make *n *decision values (binary value), and the voting strategy is utilized to make the final decision.

Random forest and data bootstrapping are implemented by Weka package [43], and default parameters are adopted.

### Performance evaluation metrics

The performance of the models is evaluated by LOOCV and the independent test. In the study, LOOCV procedure is slightly different. For a dataset of *n *structures, each time, *n*-1 structures are used to train the model, and one structure is used to test the model. In the independent test, the prediction models are trained on the training set, and then they are tested by the independent test structures.

The performance of models is scored by several metrics, i.e. sensitivity (SN), specificity (SP), F-measure (F), accuracy (ACC) and the area under ROC curve (AUC).

$$SN=\frac{\left|TP\right|}{\left|TP\right|+\left|FN\right|}$$

$$SP=\frac{\left|TN\right|}{\left|TN\right|+\left|FP\right|}$$

$$ACC=\frac{\left|TP\right|+\left|TN\right|}{\left|TP\right|+\left|TN\right|+\left|FP\right|+\left|FN\right|}$$

$$F=2*\frac{precision\times recall}{precision+recall}$$

$$precision=\frac{\left|TP\right|}{\left|TP\right|+\left|FP\right|}$$

$$recall=\frac{\left|TP\right|}{\left|TP\right|+\left|FN\right|}$$

Where *TP, TN, FP *and *FN *are the number of true positives, the number of true negatives, the number of false positives and the number of false negatives. Here, *AUC *is used as the primary evaluation metric. In order to calculate *AUC*, we use a voting cutoff to make final prediction, and then change the cutoff to obtain different *SN *and *SP*. The scores of *SN, SP, ACC *and *F *in the following tables are calculated at the cutoff that half the number of all sub-classifiers give out the positive decision.

## Results and discussions

### Performance of models based on the surface patch and thick surface patch

In order to evaluate the impact of interior residues, the surface patch-based prediction models and the thick surface patch-based models are built by combining conventional features (amino acid composition, secondary structure, conservation, and relative accessibility area). Evaluated by LOOCV, the performance of models on the bound dataset and the unbound dataset are presented in Table 1 and Table 2 respectively.

**Table 1:** Performance of the models on the bound dataset, evaluated by LOOCV

	Surface patch					Thick surface patch				
**Patch size**	**AUC**	**F**	**ACC**	**SN**	**SP**	**AUC**	**F**	**ACC**	**SN**	**SP**

12	0.609	0.217	0.712	0.412	0.748	0.618	0.220	0.713	0.407	0.750

14	0.610	0.213	0.707	0.407	0.743	0.616	0.216	0.712	0.403	0.748

16	0.614	0.218	0.720	0.410	0.759	0.620	0.219	0.722	0.404	0.765

18	0.611	0.218	0.720	0.408	0.760	0.619	0.225	0.724	0.405	0.764

20	0.612	0.220	0.729	0.398	0.771	0.621	0.226	0.731	0.403	0.774

Mean	0.611	0.217	0.718	0.407	0.756	0.619	0.221	0.720	0.404	0.760

**Table 2:** Performance of the models on the unbound dataset, evaluated by LOOCV

	Surface patch					Thick surface patch				
**Patch size**	**AUC**	**F**	**ACC**	**SN**	**SP**	**AUC**	**F**	**ACC**	**SN**	**SP**

12	0.633	0.243	0.651	0.524	0.671	0.639	0.245	0.662	0.497	0.684

14	0.631	0.230	0.658	0.477	0.684	0.640	0.247	0.671	0.497	0.696

16	0.635	0.248	0.667	0.497	0.690	0.643	0.253	0.667	0.518	0.687

18	0.636	0.235	0.657	0.466	0.683	0.644	0.246	0.658	0.505	0.680

20	0.637	0.230	0.655	0.478	0.679	0.645	0.237	0.655	0.495	0.678

Mean	0.634	0.237	0.658	0.488	0.681	0.642	0.246	0.663	0.502	0.685

In Table 1, the models based on the surface patch achieve the mean AUC value of 0.611 for the patches of different sizes (from 12 to 20 residues). The models based on the thick surface patch achieve the mean AUC value of 0.619. For different patch sizes, the thick surface patch yields consistently better results than the surface patch. As shown in Table 2, the performance enhancement can be observed on the unbound dataset, regardless of the patch size. The results indicate that the thick surface patch is likely to contain more useful information for distinguishing epitope residues from non-epitope residues.

**Figure 3:**
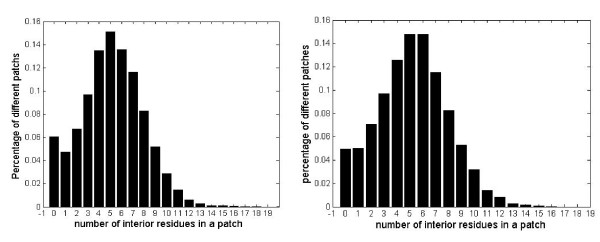
**Distribution of the composition of exterior residues in thick surface patches for the bound dataset and unbound dataset (left: bound dataset, right: unbound dataset)**.

The usefulness of interior residues in the thick surface patch is further analyzed. Figure 3 shows the relative occurrence of the 20-residue thick surface patches with different number of interior residues. It is observed that most of the thick surface patches include 4, 5, 6 or 7 interior residues. The surface residues are thought to make more contribution to the epitope prediction, given the much larger number of surface residues than interior residues in the patches.

### Performance of models using feature combination

In the section, we investigate the predictive power of the adjacent residue distance feature (ARD). In the following content, the size of patch is set to 20, for the patch size is widely used in the epitope prediction. Based on the thick surface patch, models based on individual features and their combination are built and evaluated. Table 3 presents the performance of individual features and their combination on the benchmark bound dataset and benchmark unbound dataset, evaluated by LOOCV.

**Table 3:** Performance of based models using individual feature and their combination, evaluated by LOOCV

	Bound dataset					Unbound dataset				
**Feature**	**AUC**	**F**	**ACC**	**SN**	**SP**	**AUC**	**F**	**ACC**	**SN**	**SP**

Conservation	0.554	0.186	0.634	0.418	0.660	0.558	0.188	0.604	0.445	0.634

Composition	0.563	0.163	0.747	0.275	0.801	0.566	0.147	0.708	0.244	0.774

Secondary structure	0.510	0.161	0.627	0.297	0.733	0.531	0.167	0.642	0.372	0.690

Accessible area	0.570	0.192	0.593	0.470	0.605	0.618	0.241	0.562	0.616	0.548

ARD	0.589	0.206	0.620	0.487	0.633	0.627	0.243	0.585	0.578	0.588

Combination	0.633	0.227	0.672	0.490	0.692	0.654	0.256	0.608	0.601	0.606

According to Table 3, the relative accessible area is most important for the epitope prediction among the conventional features, with the mean AUC values of 0.570 on the bound dataset and 0.618 on the unbound dataset. ARD is a useful feature that produces the mean AUC values of 0.589 on the bound dataset and 0.627 on the unbound dataset. Moreover, the combination of conventional features and ARD yields the better results than using only conventional features, with the mean AUC scores of 0.633 and 0.654 for the bound dataset and unbound dataset, respectively.

The contact number is a feature used by some existing methods. The contact number for a given residue is the number of alpha carbon atoms within a certain distance threshold (e.g. 10Å). Since the alpha carbons of the buried residues are calculated as well, the effect of interior residue is more or less considered in the contact number. The model using this individual feature can produce the mean AUC values of 0.565 and 0.619 on the bound dataset and unbound dataset, respectively. However, the contact number assigns an equal weight to every adjacent residue regardless of the distance to the central residue. The experiments are further carried out to evaluate the advantage of the thick surface patch and the adjacent residue distance feature over the contact number. As shown in Table 4, the thick surface patch-based models that combine ARD and conventional features produce better performance than the surface patch-based models that combine the contact number and conventional features. Moreover, incorporating the contact number into our model cannot make further improvement, which may be attributed to the redundant information between the contact number and the thick surface patch.

**Table 4:** The performance of our model and the model using the contact number

	Bound dataset					Unbound dataset				
**Model**	**AUC**	**F**	**ACC**	**SN**	**SP**	**AUC**	**F**	**ACC**	**SN**	**SP**

Contact Number	0.565	0.188	0.621	0.468	0.638	0.619	0.241	0.573	0.601	0.558

ARD	0.589	0.206	0.620	0.487	0.633	0.627	0.243	0.585	0.578	0.588

CF+CN	0.618	0.212	0.670	0.444	0.695	0.649	0.259	0.631	0.572	0.634

CF+ARD	0.633	0.227	0.672	0.490	0.692	0.654	0.256	0.608	0.601	0.606

CF+ARD+CN	0.623	0.215	0.654	0.481	0.6717	0.646	0.243	0.593	0.587	0.589

Contact Number and ARD mean the models using the contact number and ARD. CF+CN represents the model based on the surface patch by combining the conventional features and contact number; CF+ARD represents our model based on the thick surface patch by combining the conventional features and ARD. CF+ARD+CN means the altered edition of our method that incorporates the contact number.

### Comparing random forest with SVM and ANN

In addition to the random forest (RF), SVM and ANN are two popular machine learning methods in bioinformatics. For the purpose of comparison, SVM and ANN are used to construct the prediction models (implemented by Weka), and the default parameters are adopted. All models are construed based on the thick surface patch by combining four conventional features and ARD. As shown in Table 5, ANN-based models and SVM-based models can't yield better results than RF-based models. SVM is a state-of-the-art machine learning method, but its performance is sensitive to different values of parameters. The structure of ANN is even more complex than SVM. The parameter optimization of ANN and SVM is extremely time-consuming in the study. Since RF runs much faster than SVM and ANN (more than ten times faster in Weka) and demonstrates better performance with the default parameters, RF is used as a classification engine in the study.

**Table 5:** The performance of different machine learning methods

	Bound dataset					Unbound dataset				
**Method**	**AUC**	**F**	**ACC**	**SN**	**SP**	**AUC**	**F**	**ACC**	**SN**	**SP**

ANN	0.588	0.213	0.557	0.5832	0.548	0.627	0.245	0.532	0.668	0.505

SVM	0.595	0.213	0.543	0.606	0.533	0.637	0.253	0.541	0.688	0.523

RF	0.633	0.227	0.672	0.490	0.692	0.654	0.256	0.608	0.601	0.606

### Comparison with other methods

In recent years, several methods have been proposed to predict conformational epitopes, such as CEP [18], DiscoTope [19], PEPITO [20], ElliPro [21], SEPPA [22], Epitopia [25,26], EPCES [27], EPSVR [28] and EPMeta [28]. According to the datasets for model training, these methods can be classified into two types, methods trained on the unbound structure and methods trained on the bound structure. Generally speaking, CEP, ElliPro, SEPPA, PEPITO, DiscoTope and Epitopia are designed to predict epitopes from bound structures, while EPCES, EPSVR and EPMeta are constructed to predict epitopes from unbound structures.

First of all, we compare our method with the bound dataset-based prediction tools on the benchmark bound dataset. According to Rubinstein's work [25], CEP, DiscoTope, ElliPro and Epitopia produce the mean AUC values of 0.53, 0.62 and 0.59 and 0.6 on the benchmark bound dataset. By using the same dataset, our method produces the mean AUC value of 0.633. The results of CEP, DiscoTope, ElliPro are obtained by their servers. We notice that part of the bound dataset has been used to build these online servers; therefore some structures may be included in both training set and test set. Obviously, the results produced by the servers of CEP, DiscoTope, and ElliPro overestimated the actual performance of these methods. The results of Epitopia and our method are produced on the same dataset by the same LOOCV procedure, and the direct comparison demonstrates the superior performance of our method. Generally, our method produces better results than these benchmark methods on the bound dataset.

Further, we compare our method with the unbound dataset-based methods on the benchmark unbound dataset. Evaluated by LOOCV, our model achieves the mean AUC value of 0.654 on the benchmark dataset. As reported in [26], EPSVR and EPCES give out the mean AUC values of 0.670 and 0.644, respectively, by using the same dataset and exactly the same assessment measures. The superior performance of our method and EPSVR is attributed to the utilization of machine learning methods. Although EPSVR gives out the better result than our method, the announced result is actually the best result that EPSVR can achieve, for EPSVR adopts the SVM parameters that give out the best result of LOOCV. Therefore, our model that adopts the default parameters produces the comparable performance.

**Figure 4:**
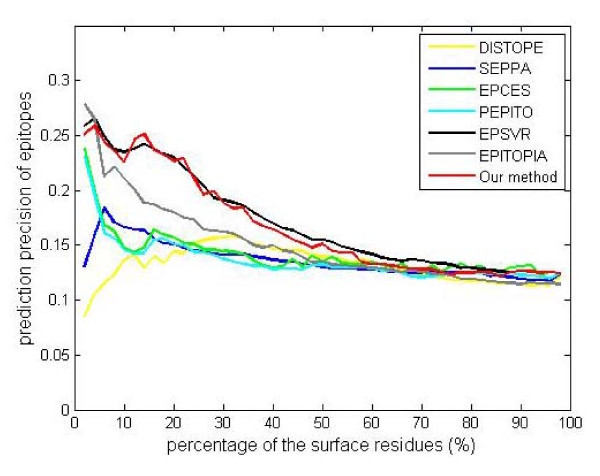
**Prediction precision of epitopes (the X axis means the percentage of surface residues that are predicted as epitopes, the prediction precision is averaged by the 19 test structures)**.

In addition, an independent test set of 19 structures are used to compare different tools and models. The mean AUC values of DiscoTope, PEPITO, SEPPA, EPITOPIA, EPCES and EPSVR calculated by their servers are 0.567, 0.570, 0.576, 0.579, 0.586 and 0.597. Our models are trained on the benchmark bound dataset and unbound dataset, respectively, and then these models are evaluated by the independent test set. The bound dataset-based model produces the mean AUC value of 0.589 for 19 structures; while the unbound dataset-based model gives out the mean AUC value of 0.598. By trained on the same bound dataset, our model produces the better result (AUC: 0.589) than EPITOPIA (AUC: 0.579) for 19 structures. By trained on the same unbound dataset, our model produces better result than EPCES (0.598 versus 0.586), and slightly better than EPSVR. EPMeta is a meta server that ensembles the results of DiscoTope, PEPITO, SEPPA, EPITOPIA, EPCES and EPSVR, and it gives out the mean AUC value of 0.638. Nevertheless, our method is better than or comparable to any independent method in the independent test. The general tendency of the prediction precision of all methods is shown in Figure 4.

We further use the paired t-test to test differences between different methods, in which the predicted AUC scores of the test structures are used. Since the statistical analysis usually requires a great number of samples, the limited number of structures in the study leads to no statistical significance (p-value > 0.05).

It is observed that the results in the independent test are significantly poorer (AUC < 0.6) than the results in the LOOCV (AUC > 0.62 for the unbound data and AUC > 0.65 for the bound data). It is not difficult to explain the performance disparity between the independent test and LOOCV. 19 independent test structures with annotated epitope sites are collected from the CED dataset [39]. In CED, the annotated epitopes sites are actually functional epitopes determined by the wet experiment. However, computational methods for the epitope prediction focus on the structural epitopes, which are determined by the distance between antigens and antibodies (or accessible surface area loss upon antibody binding). Therefore, all methods produce the relatively poor results in independent test.

**Figure 5:**
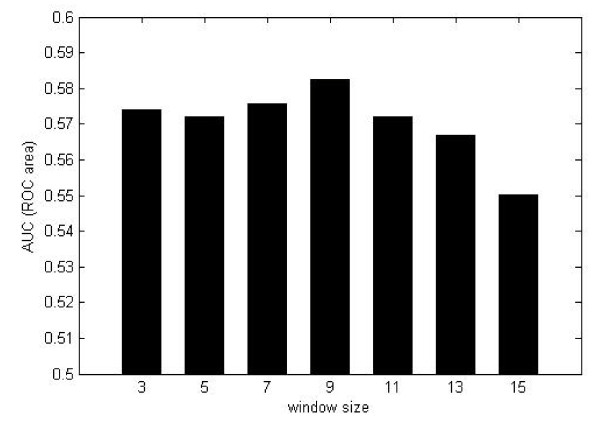
**The performance of sequence-based models with different window sizes, evaluated by LOOCV**. The accurate AUC scores in the figure are 0.574, 0.572, 0.576, 0.583, 0.572, and 0.567 for the window size from 3 residues to 15 residues.

Besides structure-based prediction methods, Raghava recently proposed a method to predict conformational epitopes from antigen sequences [30]. In the method, physicochemical features (PPP), sparse encoding scheme (BPP) and amino acid composition (CCP) are used to encode the overlapping segments from antigen sequences, and prediction models are constructed by using SVM. The model based on CCP gives out the best result; therefore, it is tested and evaluated on the benchmark sequence dataset. As shown in Figure 5, all AUC values produced by the model are less than 0.6, when the window size varies from 3 residues to 15 residues. For the corresponding structure dataset, our method produces the AUC value of 0.633.

As mentioned in the introduction, PatchDock and ClusPro can be used to predict the conformational epitopes. Differing from the methods specially designed for the conformational epitope prediction, PatchDock and ClusPro focus on the protein-protein binding site prediction. Although PatchDock and ClusPro may produce the high-accuracy performance on some bound structures [38], their power for the epitope prediction is limited by some drawbacks. Firstly, we should emphasize that, due to the different prediction purposes, PatchDock and ClusPro have to use the antibodies as well as antigens while our method is identifying the potential binding sites on the antigens when antibodies are unknown. Technically, in the conformational epitope prediction, the complexes including antigens and antibodies are used to determine the binding sites on the antigens for the purpose of labelling instances. After the binding sites are labelled on the antigens, only the antigen structures can be used to train and test the prediction models because the introduction of prior knowledge about antibodies will lead to over-estimated performance. More importantly, for these docking methods, the performance on unbound structures is quite unsatisfactory in comparison to the performance on bound structures [38]. However, the unbound structure-based prediction has more practical value. Since the proposed method only requires antigen structures to make perditions and produces satisfying results on the unbound dataset, it has superiority over the docking methods in the epitope prediction.

In general, our method demonstrates overall higher prediction accuracy on the benchmark bound dataset as well as the benchmark unbound dataset.

## Conclusions

Mining the spatial context about Ag-Ab interaction and predicting B-cell conformational epitopes are essential for understanding the immune response and vaccine design. In this study, we make a systematic investigation into the basic knowledge about epitope recognition, and aim to improve the performance of the existing methods. We develop a novel method to predict conformational epitopes based on the 'thick surface patch' by combining conventional features and the 'adjacent residue distance' feature. The experiments show that our method yields the mean AUC value of 0.633 for the benchmark bound dataset, and the mean AUC value of 0.654 for the benchmark unbound dataset, when evaluated by LOOCV. In the independent test, the bound dataset-based model and unbound dataset-based model produce the mean AUC values of 0.589 and 0.598 for 19 independent test structures, respectively. Compared with the state-of-the-art methods, our methods show comparable or better performance on the independent test set. Our study also provides biological insights into the spatial context of residues as well as the roles of conventional features in antigen-antibody interactions. The standalone tool based on the study is available at http://code.google.com/p/my-project-bpredictor/downloads/list.

## Authors' contributions

WZ designed the study, implemented the algorithm and drafted the manuscript. YX participated in the analysis and discussion, and refined the manuscript. MZ and HZ developed the prediction tool based on the study. XHY helped prepare the data and draft the manuscript. JL supervised the study and gave comments on the manuscript. All authors read and approved the final manuscript.
